# Ensemble of Domain-Specific Natural Language Processing and Large Language Models for Detecting Suicidal Ideation and Mental Health Conditions in Social Media Text: Development and Evaluation Study

**DOI:** 10.2196/98925

**Published:** 2026-10-08

**Authors:** Thomas Kallstenius, Basil Duvernoy, Adam Kallstenius, Andrea Johansson Capusan, Adam Williamson

**Affiliations:** 1 NeuroPrism AB Norrköping Sweden; 2 Center for Social and Affective Neuroscience Department of Biomedical and Clinical Sciences Linköping University Linköping, Östergötland Sweden; 3 International Clinical Research Center St. Anne’s University Hospital Brno Brno Czech Republic

**Keywords:** artificial intelligence, AI, ensemble methods, large language models, mental health informatics, natural language processing, patient safety, suicidal ideation detection, text classification

## Abstract

**Background:**

Mental illness contributes substantially to global disability, and public adoption of AI for mental health support is accelerating without commensurate safety evaluation. General-purpose large language models can fail to recognize naturalistic text expressing suicidal ideation, with immediate clinical consequences. Domain-specific natural language processing methods offer a contrasting approach, but ensembles integrating the two have not been formally characterized.

**Objective:**

We aimed to (1) benchmark 5 modeling approaches for classifying mental health–related social media text, (2) develop and optimize ensembles integrating a domain-specific natural language processing classifier with a fine-tuned large language model, and (3) derive a closed-form framework constraining ensemble weighting in safety-critical classification.

**Methods:**

We analyzed 60,889 publicly available Reddit and Twitter texts spanning 9 categories (anxiety, bipolar disorder, depression, a normal baseline, personality disorder, stress, suicidal ideation, attention-deficit/hyperactivity disorder, and autism spectrum disorder). Labels were derived from the originating subreddit or a self-stated condition and are not clinical diagnoses. Five architectures were compared: prompt-engineered base GPT-4o-mini; fine-tuned GPT-4o-mini; a domain-specific classifier (support vector machine over symptom-informed lexical features); and 2 ensembles of these models, hybrid probability–indicator fusion and soft probability fusion. Data were split into 70%, 10%, and 20% at the post level (n=42,622, n=6089, and n=12,178); weights were selected on the validation split and all reported performance comes from the held-out test split. Closed-form bounds on permissible language model weights were derived for both strategies. CIs are paired bootstrap intervals (n=10,000 replicates); accuracy differences were tested with exact McNemar tests.

**Results:**

The domain-specific classifier achieved 91.9% accuracy (95% CI 91.4%-92.4%), exceeding the base (58.7%) and fine-tuned (88.6%) large language models. Both ensembles outperformed either constituent model, reaching 93.9% (soft fusion; 95% CI 93.5%-94.3%) and 93.8% (hybrid fusion; 95% CI 93.4%-94.2%) at a weighting of 60% classifier to 40% fine-tuned model, selected on validation (*P*<.001 for both against the classifier alone; the 2 ensembles did not differ from each other [*P*=.16]). The hybrid ensemble reduced the miss rate for the suicidal ideation category from 32.5% (650/2000) to 1.9% (37/2000), a 17.6-fold reduction relative to the base model, although its advantage over the classifier alone within that category was not significant (*P*=.68). Accuracy fell discontinuously above a 50% language model weight, where the hybrid ensemble reduced exactly to the fine-tuned model; the selected weight satisfies the derived instance-level bound of 44.0%.

**Conclusions:**

Domain-specific clinical grounding remained necessary for stable ensemble performance in this corpus, and probabilistic fusion outperformed both constituent models when language model weight was bounded by a derivable, model-agnostic constraint. Because labels were community-inferred and only one corpus was analyzed, these results are hypotheses requiring external validation on clinically characterized data before use in decision support.

## Introduction

The global burden of mental illness continues to rise, with mental and substance use disorders accounting for a substantial proportion of disability-adjusted life years worldwide [1,2]. Preventive tools that support early identification of mental illness before conditions escalate remain scarce. Accurate, scalable tools to support screening, assessment, and clinical validation are urgently needed [3-5]. Public adoption of AI for mental health support is accelerating: chatbot-based interventions have demonstrated efficacy in reducing symptoms of depression and anxiety [6-9], and meta-analyses show that app-supported smartphone interventions yield meaningful effect sizes for depressive symptoms [10-12]. A growing share of younger adults now report using conversational AI for emotional support, often as a first point of contact—regardless of clinical readiness or regulatory status [13-16].

Nowhere is this gap between deployment and safety more consequential than in suicidality detection. Suicide remains one of the leading causes of preventable death globally, yet the systems being adopted most rapidly—general-purpose large language models (LLMs)—can fail to recognize the indirect, knowledge-seeking, or contextually embedded signals that characterize real-world suicidal ideation [17,18]. Transformer-based LLMs have attained fluency and general reasoning capabilities [19] that make them superficially well suited for emotional support but were never designed with clinical deployment in mind. This concern is now supported by peer-reviewed evaluations and not by preprints alone: in a controlled comparison against expert suicidologists, 3 frontier chatbots systematically overrated the appropriateness of responses to suicidal ideation [20], and in a clinician-benchmarked risk-stratification study, the same models responded inconsistently to queries of intermediate risk and diverged from clinician-assigned risk levels [21]. Across these evaluations, the recurring critical failure mode is the same: implicit suicide risk expressed in ecologically valid queries is not recognized [17].

Domain-specific natural language processing (NLP) methods, by contrast, incorporate carefully engineered features grounded in diagnostic criteria, symptom vocabularies, and validated assessment tools [22]. In our previous work, a traditional NLP classifier outperformed both prompt-engineered and fine-tuned GPT-4o-mini models on a 7-category mental health classification task [23]. That study established which single model performed best; it did not address how the 2 model families should be combined, which is the question we take up here. The present study therefore differs from our earlier work [23] in 4 respects: the label space is extended to 9 categories by adding attention-deficit/hyperactivity disorder (ADHD) and autism spectrum disorder (ASD); 2 ensemble architectures are introduced and optimized rather than single models compared; a closed-form bound is derived that explains, rather than merely reports, the observed weighting behavior; and performance is evaluated from a patient-safety perspective, with the miss rate in the suicidal ideation category as the primary safety end point. The central question is therefore not whether AI can contribute to mental health, but how models should be combined so that contextual sensitivity is gained without surrendering safety.

To address this, we investigated 5 model architectures across 60,889 mental health–related texts spanning 9 source-derived label categories, developed and optimized 2 ensemble strategies—hybrid probability–indicator fusion and soft probability fusion—and derived a mathematical framework that formally constrains LLM weight in ensemble design. Our specific aims were to quantify how much of the performance difference between the 2 model families survives ensembling, to establish whether the weighting behavior we observe is an artifact of this dataset or a structural property of the fusion rule itself, and to measure the consequences of these design choices for the detection of posts labeled as suicidal ideation. We hypothesized that a domain-specific classifier would outperform unadapted LLMs, that a weight-constrained ensemble would outperform its constituent models, and that the location of the performance cliff would be predictable from a closed-form bound rather than dataset-specific.

## Methods

### Dataset and Preprocessing

Our evaluation used a dataset of 60,889 mental health–related social media text instances across 9 categories: anxiety, bipolar disorder, depression, normal (baseline), personality disorder, stress, suicidal ideation, ADHD, and ASD. This extends our previous 7-category framework [23] by adding ADHD and ASD. The core dataset was curated from publicly available mental health datasets sourced from Reddit and Twitter (subsequently rebranded X), aggregated from the Kaggle Mental Health Sentiment Analysis dataset [23]. ADHD data were drawn from r/ADHD and r/adhdwomen; ASD data were drawn from r/autism and r/aspergers. Categories were assigned based on the originating subreddit. A stratified train-validation-test split (70%, 10%, and 20%) was applied, yielding 42,622 training, 6089 validation, and 12,178 test instances.

Dataset labels reflect explicitly stated or source-derived mental health labels and do not represent formal clinical diagnoses. A post is assigned to a category because of where it was published or what its author said about themselves, not because a clinician assessed that author. High classification accuracy on such data therefore demonstrates that a model can separate the language communities associated with these categories; it does not demonstrate that the model identifies psychiatric disorders in a diagnostic sense. An unknown but potentially substantial share of the achievable accuracy will reflect subreddit-specific register, terminology, and topic conventions rather than clinical status, and some proportion of posts in any category will have been written by people who do not have the condition the subreddit is organized around. Throughout this paper, category names such as “depression” or “suicidal ideation” should be read as labels for this discourse and not as diagnoses. The public availability and size of the corpus nevertheless make it a useful resource for investigating how NLP and LLM architectures behave in this domain.

Back-translation augmentation was applied to a 10% random sample of the corpus before partitioning, so an original statement and its back-translated variant could in principle fall on opposite sides of the split. We quantified this. No test statement appeared verbatim in the training set. Using term frequency–inverse document frequency (TF-IDF) cosine similarity, 128 of 12,178 test statements (1.1%) had a training neighbor above 0.99 similarity and 166 (1.4%) above 0.95. Classification accuracy on those 166 statements was 96.4%, against 91.8% on the remaining 12,012; excluding them changed overall accuracy from 91.9% to 91.8%. The corpus does not retain author identifiers, so partitioning was performed at the level of the post rather than the user, and we could not verify whether multiple posts by the same author spanned the split. Because users who post repeatedly within the same subreddit tend to reuse vocabulary, phrasing, and topics, the absolute accuracies reported here should be read as upper bounds on what would be obtained under strict user-level separation.

### Model Architectures

#### Domain-Specific NLP Model

We implemented a domain-specific traditional NLP classifier optimized for mental health text, using a support vector machine with radial basis function kernel and TF-IDF features (unigrams and bigrams, maximum 10,000 features) informed by symptom-specific vocabularies derived from validated diagnostic criteria. Class probabilities were obtained by Platt scaling fitted during training, and all reported classifier predictions are the argmax of that calibrated distribution; the ensemble derivations below assume these probabilities are calibrated. Data augmentation via back-translation (English to French to English) was applied to improve robustness in underrepresented classes.

#### LLM Approaches

We evaluated 2 approaches based on GPT-4o-mini (OpenAI): the general-purpose base model using prompt engineering and a version fine-tuned on mental health–specific data. For both language model configurations, class predictions and probabilities were obtained by querying the model with the instruction format used during fine-tuning and reading the log-probabilities of the first token of each of the 9 label names, which are mutually distinct under the model’s tokenizer; the resulting distribution was renormalized over the 9 classes. Applying a single elicitation procedure to every architecture is a correction relative to the submitted version of this manuscript, in which the stand-alone evaluation of the fine-tuned model reposed the task as a letter-code selection while the ensembles used the plain instruction on which that model had been fine-tuned. Because the model was fine-tuned to emit label names, the letter-code prompt was out of distribution and understated its stand-alone accuracy by approximately 14 percentage points. All results reported here use the single, consistent procedure.

### Ensemble Architectures

We evaluated 2 ensemble strategies. In hybrid probability–indicator fusion, the NLP model contributes calibrated class probabilities *P_NLP_*(*c*), while the fine-tuned LLM contributes a one-hot indicator for its predicted class *ŷ_FT_*. For each class *c*, the ensemble score is as follows:



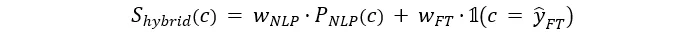



where *w_NLP_* and *w_FT_* are model weights satisfying *w_NLP_*+*w_FT_*=1. In soft probability fusion, both models contribute full probability distributions:



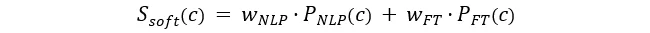



In both formulations, the final predicted class is the class c with the highest ensemble score. Weights were optimized via stratified grid search (step 0.05) on the validation split, and all reported accuracies were computed on the held-out test split, which was not used for weight selection.

### Evaluation Protocol

Five approaches were evaluated: base GPT-4o-mini, fine-tuned GPT-4o-mini, traditional NLP, hybrid probability–indicator ensemble, and soft probability ensemble. Overall accuracy served as the primary optimization criterion for ensemble weight selection, with detailed per-class analysis of precision, recall, and *F*_1_-score across all 9 categories. Weight selection used the validation split only; the test split was held out and used once for the final performance estimates reported below. All performance metrics are reported as percentages.

### Statistical Analysis

Ninety-five percent CIs for accuracy and per-class recall were obtained by bootstrap resampling of the test set with 10,000 replicates, using a common set of resamples across models so that between-model comparisons were paired. Differences in accuracy between models were tested with exact McNemar tests on discordant predictions. Because the architectures were evaluated on identical items, their errors were correlated, so differences between them should not be judged by inspecting whether their CIs overlap. Two-sided *P* values are reported; *P*<.05 was considered significant.

### Mathematical Weight Bound

Full derivations of ensemble weight bounds under binary and multiclass settings, for both hybrid and soft fusion, are provided in Multimedia Appendix 1. Two distinct quantities follow from that analysis and should not be conflated. The first is an instance-level condition: under hybrid fusion, when the NLP model correctly identifies the true class A with calibrated probability *p_A_*=*P_NLP_*(*A*) and the LLM predicts an incorrect class, the ensemble still predicts correctly if and only if *w_FT_* <1 − 1/(2*p_A_*). Evaluated at the mean calibrated confidence measured on the 917 test statements where the classifier was correct and the language model was not (*p_A_*=0.893), this gives *w_FT_* <44.0%. The second is a structural threshold that does not depend on *p_A_* at all: because the LLM contributes a one-hot indicator of magnitude *w_FT_* while the NLP contribution cannot exceed 1 − *w_FT_*, any *w_FT_* >0.5 makes the LLM’s predicted class the argmax for every input, so the hybrid ensemble becomes identical to the fine-tuned LLM. The instance-level bound describes where the optimum lies; the structural threshold explains why the accuracy curve is discontinuous there. Both depend only on the calibration of the probabilistic classifier and on the hard, one-hot form of the LLM output, not on the identity of either model.

### Ethical Considerations

This study analyzed only publicly available, aggregated social media text that had already been collected, deidentified, and released as a research dataset by third parties. No individuals were contacted, no private or access-restricted content was obtained, and no attempt was made to reidentify authors or to link posts to accounts. The work therefore does not constitute research on human participants within the meaning of the Swedish Act concerning the Ethical Review of Research Involving Humans (2003:460), and no ethical review board approval or waiver was required. Because the corpus nevertheless consists of first-person disclosures about mental health, no verbatim post text is reproduced in this paper, and no derived data that could support reidentification are released.

## Results

### Individual Model Performance

Evaluation revealed substantial performance differences across modeling approaches (Figure 1). The base LLM achieved 58.7% overall accuracy (95% CI 57.9%-59.6%), with an *F*_1_-score for personality disorder of only 16% and a recall of 67.5% in the suicidal ideation category, meaning that 1 in 3 posts carrying that label was not identified. The base LLM’s *F*_1_-scores for stress (27%) and ADHD (49%) were also low. Fine-tuning improved performance to 88.6% (95% CI 88.1%-89.2%), but this did not reach the domain-specific NLP model, which achieved 91.9% (95% CI 91.4%-92.4%) with per-condition *F*_1_-scores ranging from 78% (ADHD) to 99% (personality disorder). The 2 model families showed different strengths in terms of *F*_1_-score: the classifier led on personality disorder (99% vs 89%), stress (97% vs 84%), and suicidal ideation (97% vs 89%), whereas the fine-tuned model led on ADHD (86% vs 78%). Per-condition precision, recall, and *F*_1_-score with 95% CIs for recall are given in Multimedia Appendix 2.

**Figure 1:**
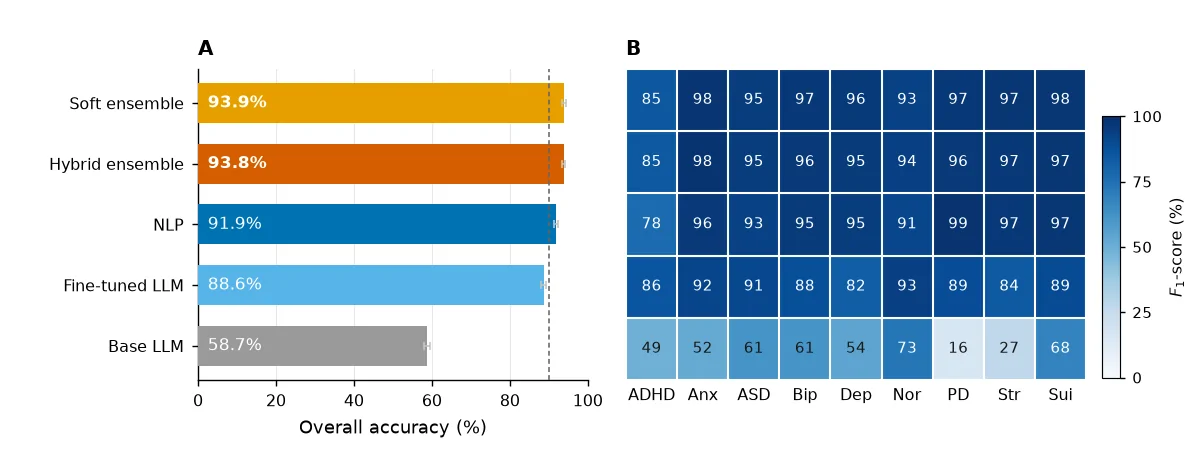
Overall accuracy and per-condition *F*_1_-score across 5 modeling approaches (held-out test set, n=12,178). (A) Overall accuracy with 95% CIs; the dashed line marks 90% accuracy. (B) *F*_1_-score by condition and model, with rows aligned with the models in panel A. ADHD: attention-deficit/hyperactivity disorder; Anx: anxiety; ASD: autism spectrum disorder; Bip: bipolar disorder; Dep: depression; LLM: large language model; NLP: natural language processing; Nor: normal; PD: personality disorder; Str: stress; Sui: suicidal ideation.

### Confusion Matrices: Pattern of Errors

Figure 2 compares row-normalized confusion matrices for the base LLM, NLP model, and optimized hybrid ensemble. The base LLM showed severe off-diagonal errors, particularly depression cases misclassified as anxiety (12.5%) and personality disorder cases widely scattered. The NLP model achieved near-diagonal classification across all 9 conditions, with the main residual error being ADHD recall (66.1%), with 24.7% of ADHD statements being classified as normal, reflecting linguistic similarity between ADHD self-reports and other categories. The hybrid ensemble further improved performance, raising ADHD recall to 75.0%, reducing ADHD-to-normal confusion from 24.7% to 17.6%, and reaching 98.2% recall in the suicidal ideation category.

**Figure 2:**
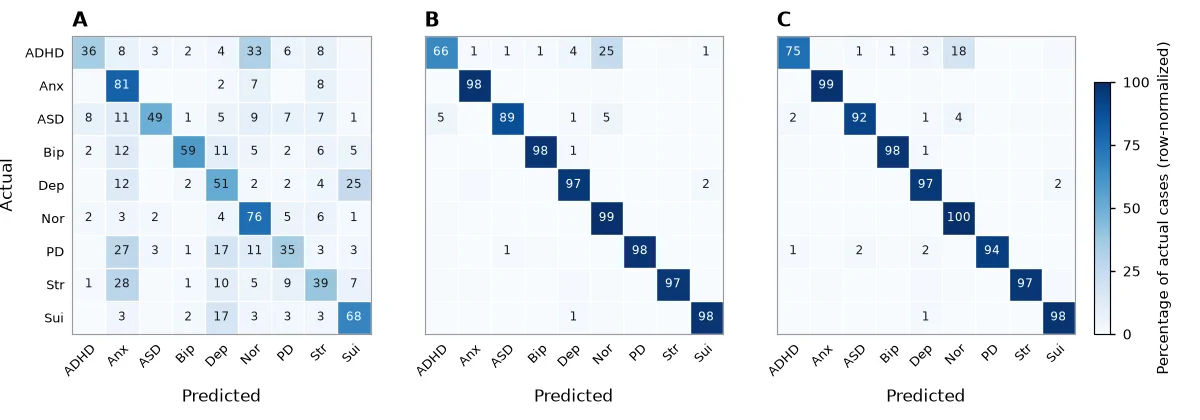
Row-normalized confusion matrices for the base large language model (LLM), the domain-specific natural language processing (NLP) classifier, and the hybrid probability–indicator ensemble (held-out test set, n=12,178). All values are percentages of actual cases within each row. (A) Base LLM (58.7% accuracy). (B) NLP classifier (91.9%). (C) Hybrid ensemble (93.8%). ADHD: attention-deficit/hyperactivity disorder; Anx: anxiety; ASD: autism spectrum disorder; Bip: bipolar disorder; Dep: depression; Nor: normal; PD: personality disorder; Str: stress; Sui: suicidal ideation.

### Ensemble Optimization

Figure 3 shows the weight optimization curves for both ensemble strategies. Weights were selected on the validation split; both ensembles selected *w_FT_*=40%, giving test accuracies of 93.8% for the hybrid ensemble and 93.9% for the soft ensemble. Both ensembles exceeded the classifier alone, by 1.9 and 2.0 percentage points, respectively (*P*<.001 for each), and their gain over the classifier was concentrated in ADHD (*F*_1_-score 85% vs 78%), the classifier’s weakest category. The optimism avoided by selecting on a separate split is small: the best test-split value for either ensemble exceeds the value at the selected weight by 0.1 percentage points to 0.2 percentage points. Beyond *w_FT_*=50% the hybrid ensemble dropped abruptly to 88.6% and remained constant, because once the language model weight exceeded one-half, the indicator term necessarily dominated the probabilistic term for every instance, and the ensemble reduced exactly to the fine-tuned model; the flat branch therefore lay at the fine-tuned model’s own accuracy and was analytically determined rather than empirically estimated (Multimedia Appendix 1). The soft ensemble degraded gradually to the same end point, since both of its terms remained probabilistic. Both curves confirmed that language model authority must remain below 50% of ensemble weight for the hybrid architecture to retain any contribution from the classifier at all.

**Figure 3:**
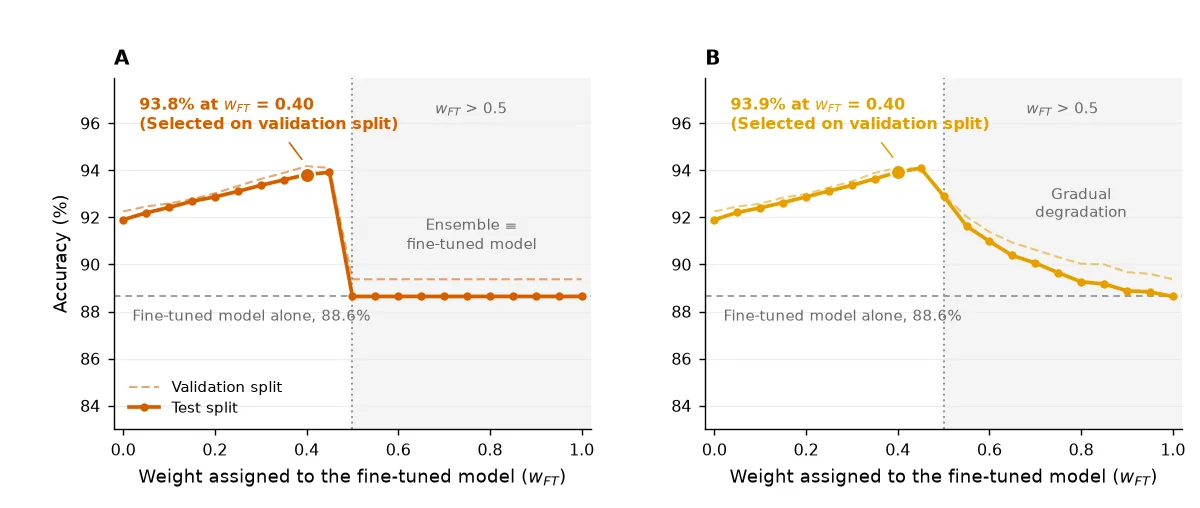
Ensemble weight optimization curves. Accuracy is plotted against the weight assigned to the fine-tuned model (wFT). The solid line is the held-out test split (n=12,178) and the dashed line is the validation split (n=6089) on which the weight was selected; the marked point is the selected weight. (A) Hybrid probability–indicator fusion. (B) Soft probability fusion.

### Safety Recall by Condition

Figure 4 reports recall by condition. Recall in the suicidal ideation category is the most clinically consequential quantity we measure, subject to the caveat that the category is defined by subreddit membership rather than by clinical assessment: the base LLM missed 32.5% (650/2000) of the posts carrying this label; the fine-tuned LLM missed 9.1% (181/2000; recall 91.0%); the NLP model missed 2% (40/2000; recall 98.0%); and the hybrid ensemble missed 1.9% (37/2000; recall 98.2%). This 17.6-fold reduction in miss rate between the base LLM and the hybrid ensemble is large enough to matter in any screening application, although its clinical value can only be established on clinician-labeled data. The ensemble achieved the highest recall for ADHD (75.0%, against 66.1% for the classifier alone). ADHD was the only category in which the ensemble remained below 90% recall. The difference in suicidal ideation recall between the hybrid ensemble and the classifier alone was not statistically significant (98.2% vs 98.0%; *P*=.68); the safety gain was established against the base language model (*P*<.001), not against the domain-specific classifier.

**Figure 4:**
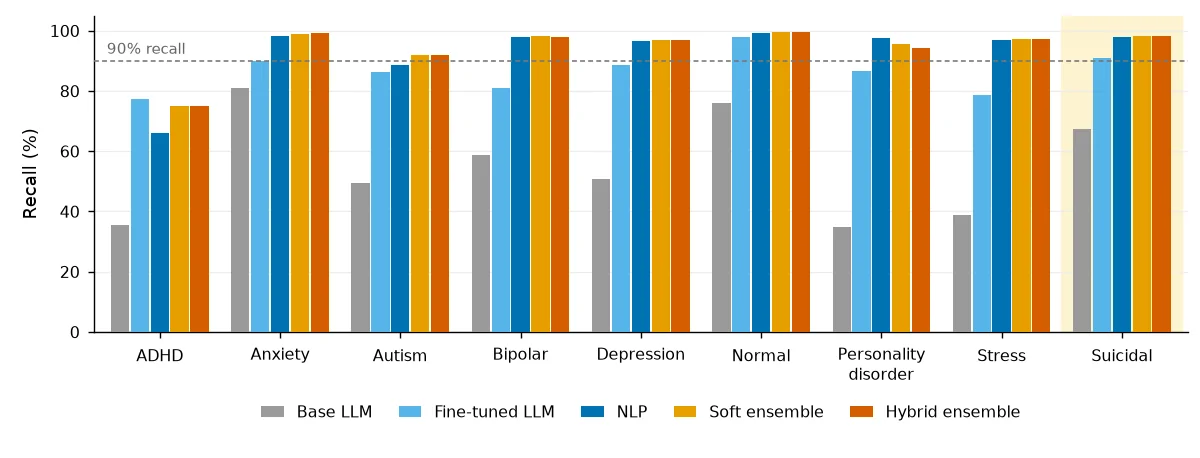
Recall by mental health category and model (held-out test set, n=12,178). Each cluster shows 1 of 9 categories, defined by subreddit membership rather than by clinical assessment; the dashed horizontal line marks 90% recall, and the shaded column is the suicidal ideation category. ADHD: attention-deficit/hyperactivity disorder; LLM: large language model; NLP: natural language processing.

## Discussion

### Domain Expertise as a Design Constraint

Our results indicate that domain-specific expertise should remain central to mental health AI systems of this kind. The NLP model’s higher individual accuracy (91.9% vs 88.6%) is modest in aggregate, but the per-condition picture is sharper: the classifier holds a clear advantage on the categories with the least training data and the greatest clinical consequence, including personality disorder and suicidal ideation, while the fine-tuned model is stronger on ADHD. The ensemble optimization results make the structural point more directly than the accuracy comparison does: the selected configuration gives the classifier majority control (60%), and performance degrades sharply the moment that ordering is reversed. That bound is derived rather than tuned and does not depend on the size of the accuracy gap between the 2 models. We note, however, that this conclusion is drawn from a single corpus with community-inferred labels and that the advantage of engineered features may be smaller on data whose categories are not aligned with distinct online communities.

### Mathematical Analysis of Ensemble Constraints

To explain the shape of the curves in Figure 3A, we derived upper bounds on LLM weight under both ensemble formulations (full derivations in Multimedia Appendix 1). In binary classification, when the NLP model correctly identifies the true class A with calibrated probability *p_A_*=*P_NLP_*(*A*) while the LLM predicts the incorrect class B, the hybrid ensemble predicts correctly if and only if *w_FT_* < 1 − 1/(2*p_A_*). Since *p_A_* > 0.5, this bound is strictly less than 0.5. Evaluated at *p_A_*=0.893, measured on the 917 statements where the classifier was correct and the language model was incorrect, the bound yields *w_FT_* < 44.0%. The weight selected on the validation split, 40%, satisfies this bound, and the accuracy discontinuity occurs at exactly 50% on both the validation and test splits. In the multiclass case, the corresponding condition is *w_FT_*< (*p_A_* − *p_B_*)/(1 + *p_A_* − *p_B_*), where *p_B_* is the NLP probability of the class the LLM has chosen; this reduces to the binary bound when *p_B_* = 1 − *p_A_* and is stricter whenever the LLM’s error targets a class that the NLP model already considers plausible. Separately from this instance-level condition, the hybrid rule has a hard structural limit at *w_FT_*=0.5, beyond which the one-hot term alone determines the argmax and the ensemble collapses onto the fine-tuned LLM irrespective of *p_A_*; this is the discontinuity visible in Figure 3A. The soft probability fusion bound is generally looser, which explains the gradual degradation in Figure 3B. Two caveats limit the reach of these results. They are conditions on the disagreement case under a specific pair of fusion rules, not a universal design principle for classifier combination, and they assume the probabilistic classifier is calibrated, so systematic overconfidence or underconfidence will displace the bound. Where calibration cannot be verified, the safe reading is the structural limit at 0.5 rather than the tighter *p_A_*-dependent value.

We note that the bound governs whether an individual disagreement is resolved correctly, whereas Figure 3 plots aggregate accuracy; their agreement is an empirical result rather than an identity.

### Ensemble Synergy and Clinical Implications

The recall analysis in Figure 4 shows the practical consequence of model choice. The base LLM’s 32.5% miss rate in the suicidal ideation category is approximately 17.6 times the hybrid ensemble’s 1.9%. These gains arise from partial error complementarity: of the 1904 statements misclassified by at least 1 of the 2 constituent models, 1437 (75.5%) were classified correctly by the other. The errors are not independent, however—both models failed on 467 (3.8%) statements, against 0.9% expected under independence—indicating a core of intrinsically ambiguous text that neither approach resolves. An oracle that always deferred to whichever model was correct would therefore reach 96.2%, so the 93.8% achieved by the hybrid ensemble leaves roughly 2.5 percentage points of headroom, and no weighting of these 2 models can exceed the oracle. Comparing the 2 fusion strategies, soft fusion attains nominally higher accuracy but the difference is not significant (*P*=.16); we prefer the hybrid formulation on the grounds that its weight admissibility condition is a hard structural property that can be checked before deployment, not because it is more accurate.

### Comparison With Prior Work

The strongest comparator for this study is our own earlier 7-category evaluation [23], and the relationship between the two deserves to be stated precisely. The core qualitative finding—that a domain-specific classifier outperforms both prompt-engineered and fine-tuned GPT-4o-mini on this family of corpora—was established there and is reproduced, not discovered, here, and the margin is now considerably narrower: 3.3 percentage points on 9 categories against 4 points on 7 categories. What is new is the ensemble analysis: 2 fusion architectures, a closed-form account of why their weight response takes the form it does, an explicit structural threshold at which hybrid fusion degenerates, and a safety-oriented evaluation centered on the miss rate in the suicidal ideation category. The 2 additional categories (ADHD and ASD) are a secondary contribution, and they behave differently from one another: ADHD is the hardest of the 9 categories for the classifier and for both ensembles, whereas ASD is handled comparatively well.

The submitted version of this manuscript reported the fine-tuned model at 74.5% accuracy, an unexplained regression relative to the 91% reported in our earlier study. That figure was an artifact of the elicitation mismatch described in the Methods section and is corrected here to 88.6%. We record the discrepancy explicitly because it changes how the present results should be read: on the corrected measurement, the 2 model families are much closer in aggregate accuracy than the submitted version implied, and the case for domain-specific grounding rests on the derived weight bound and on per-condition performance in the safety-critical categories rather than on a large aggregate gap. The ensembles’ advantage over the classifier alone, which is the paper’s principal empirical claim, is unaffected by the correction and is now larger rather than smaller.

### Ethical Considerations and Deployment Safeguards

The results above concern classification accuracy, which is a necessary but not sufficient condition for responsible use. Three requirements follow directly from our findings. First, any deployment must be a screening aid that routes text to a human reviewer, never an autonomous triage or diagnostic decision; the labels this system was trained on are not diagnoses, and the system has never been evaluated against clinician judgment. Second, false positives are not cost-free. A system that flags suicidal ideation imposes a burden on the person flagged and on the service reviewing the flag, and any deterministic safety layer placed above the ensemble trades specificity for sensitivity by design; the resulting false-positive rate must be measured in the target population, monitored after deployment, and reported to the clinicians who act on the output. Third, because the optimal weight range is narrow and calibration drifts as language use and model versions change, continuous monitoring with scheduled recalibration and a documented rollback path is a condition of safe operation rather than an optional extra. Governance arrangements should also address consent and secondary use: individuals who post in support communities did not write for the purpose of training classifiers, and any prospective system operating on identifiable content requires an explicit legal and ethical basis of its own.

### Limitations

Several limitations constrain the generalizability and clinical applicability of our findings. First, and most importantly, the dataset consists of social media text with community-inferred rather than clinician-validated labels, so the task we solve is the identification of mental health–related discourse and not the detection of psychiatric disorders; the gap between the two is large and is not quantified by any metric reported here. Second, back-translation augmentation preceded partitioning, and partitioning was performed at the post rather than the user level; the near-duplicate overlap this creates is quantified in the Methods section, but account-level leakage cannot be excluded because the corpus does not retain author identifiers. Third, all experiments were conducted on a single aggregated corpus, and no external validation was performed; the optimal weights, the measured calibrated confidence, and hence the numerical value of the derived bound may not transfer to clinical note formats, structured assessments, or other cultural and linguistic contexts. Fourth, the ensemble’s advantage over the classifier alone in the safety-critical category is not statistically significant (*P*=.68), so the safety case rests on the comparison with the base language model. Fifth, we did not evaluate clinical utility metrics such as screening effectiveness or treatment outcomes. Sixth, the narrow optimal weight range implies that any deployment would require ongoing monitoring and adaptive recalibration. Prospective validation in clinically diagnosed populations remains an essential prerequisite before clinical use.

### Conclusions

Integrating a domain-specific NLP model into an LLM ensemble architecture improved both accuracy and safety in the classification of mental health–related social media text. The optimized ensembles achieved 93.8% (hybrid) and 93.9% (soft) overall accuracy, each exceeding both constituent models, and reduced the miss rate in the suicidal ideation category 17.6-fold relative to a general-purpose LLM baseline. We further provide a closed-form bound showing why the language model weight must remain below approximately 44% under hybrid fusion at the calibrated confidence measured here and why the architecture degenerates entirely above 50%, converting an empirical observation into a design constraint that can be checked in advance. On this evidence, domain-specific clinical grounding is best regarded as a necessary component of safe mental health text classification rather than an optional refinement. Because the evaluation rests on a single corpus with community-inferred labels, the wider claim—that these constraints transfer to other biomedical informatics applications in which model failures carry direct safety consequences—is offered as a hypothesis for external validation rather than as a demonstrated result.

## Appendix

Multimedia Appendix 1 Mathematical analysis of ensemble weight bounds—derivations for hybrid probability–indicator fusion and soft probability fusion in the binary and multiclass settings, and a proof that hybrid fusion degenerates to the fine-tuned language model for wFT≥0.5.

Multimedia Appendix 2 Per-condition precision, recall, and *F*_1_-score for all 5 architectures on the held-out test split, with 95% bootstrap CIs for recall and per-condition test set sizes.

## Abbreviations

ADHD: attention-deficit/hyperactivity disorder
ASD: autism spectrum disorder
LLM: large language model
NLP: natural language processing

## Data Availability

The aggregated corpora analyzed in this study are publicly available: the core dataset is the Kaggle Mental Health Sentiment Analysis dataset, and the attention-deficit/hyperactivity disorder (ADHD) and autism spectrum disorder (ASD) material was drawn from the public subreddits r/ADHD, r/adhdwomen, r/autism, and r/aspergers. The per-model outputs underlying every figure in this paper, including the validation and test weight sweeps and the per-condition confusion matrices, are available from the corresponding author on request.
