# Supplementary material for: Ensemble of Domain-Specific Natural Language Processing and Large Language Models for Detecting Suicidal Ideation and Mental Health Conditions in Social Media Text: Development and Evaluation Study
Source: JMIR Ment Health. 2026 Oct 8;13:e98925. doi: 10.2196/98925 (PMC13691473; doi:10.2196/98925)
Supplement: Supplementary file 1 [file mental_v13i1e98925_app1.docx]

*Supplementary material for the manuscript “Ensemble of Domain-Specific Natural Language Processing and Large Language Models for Detecting Suicidal Ideation and Mental Health Conditions in Social Media Text: Development and Evaluation Study” by Kallstenius et al.*

Multimedia Appendix 1. Mathematical analysis of ensemble weight bounds

*Part 1 – Hybrid Probability–Indicator Fusion*

**Overview.**

To explain the sharp accuracy peak observed in Fig. 3(a), we analyse the ensemble behaviour in a simplified mathematical framework. In particular, we focus on the disagreement case in which the domain-specific NLP classifier predicts the correct class while the fine-tuned LLM predicts an incorrect class. This scenario is analytically important because it represents the critical failure mode for the ensemble: if the LLM is given too much weight, it can override a correct NLP prediction and degrade overall accuracy.

This focus is also empirically justified. In our evaluation, the domain-specific NLP model demonstrates substantially higher standalone accuracy than the LLM, meaning that disagreements between the two models are more likely to correspond to LLM errors than NLP errors. Analysing this configuration therefore provides a conservative upper bound on the allowable LLM contribution. If the ensemble remains correct under this disagreement scenario, it will also remain correct when both models predict the same class, including the case where the LLM prediction is also correct.

The following derivations therefore formalise the conditions under which the ensemble prediction remains aligned with the correct NLP classification despite an incorrect LLM output, and clarify why the LLM must never dominate the ensemble weighting under the hybrid probability–indicator formulation.

**Binary case.**

Consider a binary classification setting with two classes: A (the true mental health label) and B (representing the aggregated set of incorrect labels). Let the domain-specific NLP model assign a calibrated probability $P_{NLP}\left( A \right)=\hat{p}_{A}$,$P_{NLP}\left( B \right)=1-\hat{p}_{A}$, with $\hat{p}_{A}\in]50\%,100\%]$. Let $ŷ_{FT}\in\{A, B\}$ denote the class label predicted by the fine-tuned LLM, and assume the LLM predicts the incorrect class ($ŷ_{FT}=B$). In the hybrid ensemble, the LLM contributes a hard decision—a score of $1$ for its predicted class and $0$ for all others.

For a class $i$, the ensemble scoring function is:

|  | $S(i)= w_{NLP}.P_{NLP}\left( i \right)+w_{FT}\mathbb{. 1}\left( i = ŷ_{FT} \right),$ | $where w_{NLP}+w_{FT}=1$ |
| --- | --- | --- |

Hence, the ensemble scores for the two classes are:

$$S_{A}=(1-w_{FT}).\hat{p}_{A}$$

and

$$s_{B}=(1-w_{FT}).\left( 1-\hat{p}_{A} \right)+w_{FT}$$

Requiring $s_{A}>s_{B}$ and solving for $w_{FT}$ yields the binary upper bound:

$$w_{FT}<1-\frac{1}{2.\hat{p}_{A}}$$

Since $\hat{p}_{A}\in]50\%, 100\%]$, then $(1-1/{\left( 2.\hat{p}_{A} \right))}\in]0, \frac{1}{2}]$, restricting the highest upper boundary of $w_{FT}$ to $50\%$. The LLM can therefore never dominate the ensemble when the NLP model is more likely correct. Several key insights follow:

**Bounded contribution.** Since $\hat{p}_{A}>50\%$, the bound $w_{FT}<1-1/{(2.\hat{p}_{A})}$ is always strictly less than 50%. The LLM can never dominate.

**Asymptotic convergence.** As $\hat{p}_{A}\to100\%$, the bound approaches $50\%$. Even a perfectly confident NLP model allows at most a $50\%$ LLM contribution.

**Empirical alignment.** With $\hat{p}_{A}\approx90\%$ (estimated from our data), the bound yields $w_{FT}<44\%,$ closely matching the empirically optimal $40-45\%$ in Fig. 3(a).

**Model-agnostic constraint.** The bound depends only on the NLP model’s confidence and the hard, one-hot nature of the LLM output. It applies to any ensemble combining a calibrated probabilistic classifier with a hard decision rule.

**Multiclass extension.**

In the multiclass setting with class set $C$, true class $A$, and LLM-predicted incorrect class $B\neq A$, let the NLP model assign probabilities $\{C_{i}\}$ satisfying $\hat{p}_{A}>\hat{p}_{i}$ for all $i\neq A$. The ensemble scores for the relevant classes are:

$$S_{A}=(1-w_{FT}).\hat{p}_{A}$$

and

$$S_{B}=\left( 1-w_{FT} \right).\hat{p}_{B}+w_{FT}$$

For all other classes $C\notin\{A, B\}$, $S_{C}=\left( 1 - w_{FT} \right).C_{C}\leq S_{A}$ (since $C_{A}$ is maximal). Hence the ensemble is correct if and only if $S_{A}>S_{B}$, which yields the multiclass upper bound:

$$w_{FT}<\frac{\hat{p}_{A}-\hat{p}_{B}}{1+ \hat{p}_{A}-\hat{p}_{B}}$$

In the binary case, where $\hat{p}_{B}=1-\hat{p}_{A}$, this reduces exactly to the binary bound. Unlike the binary setting, the multiclass bound depends explicitly on the gap $\hat{p}_{A}-\hat{p}_{B}$ between the true class probability and the probability of the specific incorrect class selected by the LLM. Conditions where the LLM’s error targets a class already close in NLP probability to the true class require a stricter LLM weight constraint.

*Part 2 – Soft Probability Fusion*

In soft probability fusion, both classifiers contribute full probability distributions combined through weighted linear fusion:

| $S_{soft}\left( i \right)= w_{NLP}.P_{NLP}\left( i \right)+w_{FT} .P_{FT}\left( i \right)$, | $where w_{NLP}+w_{FT}=1$ |
| --- | --- |

**Binary case.**

With $P_{NLP}\left( A \right)>P_{FT}\left( A \right)$ (NLP model is more accurate on class A), requiring $S_{soft}(A)>S_{soft}(B)$ yields:

$$w_{FT}<\frac{2.P_{NLP}\left( A \right)-1}{2.(P_{NLP}\left( A \right)-P_{FT}\left( A \right))}$$

When the LLM is confidently wrong ($P_{FT}\left( A \right)\approx0$), this bound reduces to the hybrid fusion bound. In general the soft bound is looser: even an incorrect LLM prediction may still assign non-negligible probability to the true class, partially mitigating the error. This explains the shallower degradation curve in Fig. 3(b).

**Multiclass generalisation.**

Let A be the true class and B the incorrect class receiving the highest LLM probability. Writing PNLP(A) and PNLP(B) for the NLP probabilities of these two classes and PFT(A) and PFT(B) for the corresponding LLM probabilities, the multiclass soft fusion bound is:

$$w_{FT}<\frac{P_{NLP}\left( A \right)-P_{NLP}\left( B \right)}{\left( P_{NLP}\left( A \right)-P_{NLP}\left( B \right) \right)+\left( P_{FT}\left( B \right)-P_{FT}\left( A \right) \right)}$$

This generalises the binary case and shows that correctness depends jointly on the separation of NLP probabilities and the extent to which the LLM reallocates probability mass toward an incorrect class.

*Part 3 – Comparison of Hybrid and Soft Fusion*

Under hybrid fusion, the LLM contributes a one-hot vote. The bound $w_{FT}<1-1/{(2.\hat{p}_{A})}$ is always strictly less than 50% for $\hat{p}_{A}\in]50\%,100\%]$. The LLM must never dominate under hybrid fusion.

Under soft fusion, the bound coincides with the hybrid case only when the LLM is confidently wrong. When the LLM assigns non-negligible probability to the true class, soft fusion permits substantially larger $w_{FT}$, and the bound may exceed $50\%$. This difference explains the greater numerical stability and broader range of effective weight settings observed empirically for soft fusion, compared to the sharply peaked optimum of the hybrid ensemble (Fig. 3(a) vs Fig. 3(b)).

*Key implication:* For safety-critical applications where LLM failures tend to be hard and confident (e.g., suicidal ideation misclassification), hybrid fusion with a strict $w_{FT}<44\%$ constraint is the preferred design choice. Soft probability fusion is more robust to small LLM errors and tolerates a wider range of weights, but sacrifices the hard boundary guarantee provided by the hybrid formulation.

*Part 4 – Degeneracy of Hybrid Fusion for w_FT_ ≥ 0.5*

The hybrid scoring rule assigns to each class c the score S(c) = w_NLP_·P_NLP_(c) + w_FT_·𝟙(c = ŷ_FT_), with w_NLP_ + w_FT_ = 1 and P_NLP_ a probability distribution over the class set. Consider the class ŷ_FT_ predicted by the fine-tuned LLM and any other class c ≠ ŷ_FT_. Then S(ŷ_FT_) ≥ w_FT_, because the NLP term is non-negative, while S(c) = w_NLP_·P_NLP_(c) ≤ w_NLP_ = 1 − w_FT_, because P_NLP_(c) ≤ 1. If w_FT_ > 0.5 then w_FT_ > 1 − w_FT_, so S(ŷ_FT_) > S(c) for every c ≠ ŷ_FT_ and for every input, whatever the NLP model predicts and however confident it is.

Two consequences follow. First, for any w_FT_ > 0.5 the hybrid ensemble reproduces the fine-tuned LLM’s prediction on every item, so its accuracy is exactly the accuracy of the fine-tuned LLM on the same data; the accuracy curve is flat on that interval and its level is fixed by a single measured quantity rather than estimated from the sweep. This is what Fig. 3(a) of the main text shows, where the flat branch lies at 88.6%, the fine-tuned model’s own test accuracy. Second, the transition is a step and not a gradual decline. Writing m(x) = max_c≠ŷ_ P_NLP_(c) − P_NLP_(ŷ_FT_) for the NLP margin between its preferred class and the LLM’s choice on item x, the ensemble follows the NLP model exactly when m(x) > w_FT_/(1 − w_FT_) and the LLM otherwise. The right-hand side increases from 0 to 1 as w_FT_ increases from 0 to 0.5, so the fraction of items on which the LLM overrides a confident NLP prediction grows monotonically over that interval and reaches unity at w_FT_ = 0.5, since m(x) ≤ 1 by construction.

This is a property of the fusion rule rather than of the dataset: any hybrid combination of a normalised probabilistic classifier with a hard one-hot vote behaves this way. It also distinguishes hybrid from soft fusion, where the LLM contributes a distribution rather than an indicator, no single term can dominate for structural reasons, and convergence on the LLM occurs only in the limit w_FT_ → 1.
