# Supplementary material for: Ensemble of Domain-Specific Natural Language Processing and Large Language Models for Detecting Suicidal Ideation and Mental Health Conditions in Social Media Text: Development and Evaluation Study
Source: JMIR Ment Health. 2026 Oct 8;13:e98925. doi: 10.2196/98925 (PMC13691473; doi:10.2196/98925)
Supplement: Supplementary file 2 [file mental_v13i1e98925_app2.docx]

**Multimedia Appendix 2**

Per-condition precision, recall, and *F*_1_-score for all 5 architectures on the held-out test split, with 95% bootstrap CIs for recall and per-condition test set sizes.

**Table 1.** Per-condition test set sizes (n=12,178).

| **Category** | **Test set, n (%)** |
| --- | --- |
| Attention-deficit/hyperactivity disorder | 2000 (16.4) |
| Anxiety | 716 (5.9) |
| Autism spectrum disorder | 1242 (10.2) |
| Bipolar disorder | 500 (4.1) |
| Depression | 2000 (16.4) |
| Normal | 3085 (25.3) |
| Personality disorder | 178 (1.5) |
| Stress | 457 (3.8) |
| Suicidal ideation | 2000 (16.4) |
| **Total** | **12,178 (100)** |

**Table 2.** Per-condition performance of each architecture on the held-out test split.

| **Architecture and category** | **Precision, %** | **Recall, % (95% CI^a^)** | ***F*_1_-score, %** |
| --- | --- | --- | --- |
| **Base LLM** | | | |
| Attention-deficit/hyperactivity disorder | 79.9 | 35.5 (33.4-37.6) | 49.1 |
| Anxiety | 37.8 | 81.0 (78.1-83.8) | 51.6 |
| Autism spectrum disorder | 80.8 | 49.4 (46.7-52.3) | 61.3 |
| Bipolar disorder | 63.7 | 58.6 (54.3-63.0) | 61.0 |
| Depression | 57.7 | 50.8 (48.7-53.0) | 54.0 |
| Normal | 70.1 | 76.1 (74.6-77.6) | 73.0 |
| Personality disorder | 10.6 | 34.8 (27.8-42.1) | 16.3 |
| Stress | 21.1 | 39.0 (34.5-43.4) | 27.3 |
| Suicidal ideation | 67.8 | 67.5 (65.4-69.5) | 67.6 |
| **Fine-tuned LLM** | | | |
| Attention-deficit/hyperactivity disorder | 96.2 | 77.3 (75.5-79.1) | 85.7 |
| Anxiety | 93.6 | 89.9 (87.7-92.1) | 91.7 |
| Autism spectrum disorder | 95.6 | 86.2 (84.2-88.1) | 90.6 |
| Bipolar disorder | 96.4 | 81.0 (77.4-84.4) | 88.0 |
| Depression | 76.8 | 88.8 (87.4-90.1) | 82.4 |
| Normal | 89.3 | 98.0 (97.5-98.5) | 93.4 |
| Personality disorder | 91.1 | 86.5 (81.3-91.4) | 88.8 |
| Stress | 90.2 | 78.6 (74.7-82.3) | 84.0 |
| Suicidal ideation | 87.4 | 91.0 (89.7-92.2) | 89.1 |
| **NLP classifier** | | | |
| Attention-deficit/hyperactivity disorder | 94.0 | 66.1 (64.0-68.2) | 77.6 |
| Anxiety | 94.0 | 98.3 (97.3-99.2) | 96.1 |
| Autism spectrum disorder | 97.4 | 88.6 (86.8-90.4) | 92.8 |
| Bipolar disorder | 93.0 | 98.0 (96.7-99.2) | 95.4 |
| Depression | 93.8 | 96.7 (95.9-97.5) | 95.2 |
| Normal | 84.1 | 99.3 (99.0-99.6) | 91.1 |
| Personality disorder | 99.4 | 97.8 (95.3-99.5) | 98.6 |
| Stress | 96.7 | 96.9 (95.3-98.4) | 96.8 |
| Suicidal ideation | 96.5 | 98.0 (97.4-98.6) | 97.3 |
| **Soft probability ensemble** | | | |
| Attention-deficit/hyperactivity disorder | 97.5 | 75.0 (73.2-77.0) | 84.8 |
| Anxiety | 96.9 | 99.0 (98.2-99.7) | 97.9 |
| Autism spectrum disorder | 97.5 | 92.0 (90.4-93.5) | 94.7 |
| Bipolar disorder | 95.0 | 98.4 (97.2-99.4) | 96.7 |
| Depression | 94.4 | 97.0 (96.3-97.8) | 95.7 |
| Normal | 87.8 | 99.5 (99.3-99.7) | 93.3 |
| Personality disorder | 98.3 | 95.5 (92.2-98.3) | 96.9 |
| Stress | 97.2 | 97.4 (95.8-98.7) | 97.3 |
| Suicidal ideation | 96.7 | 98.3 (97.7-98.8) | 97.5 |
| **Hybrid probability–indicator ensemble** | | | |
| Attention-deficit/hyperactivity disorder | 97.3 | 75.0 (73.2-77.0) | 84.8 |
| Anxiety | 97.0 | 99.2 (98.4-99.7) | 98.1 |
| Autism spectrum disorder | 97.2 | 92.0 (90.4-93.5) | 94.5 |
| Bipolar disorder | 94.9 | 97.8 (96.4-99.0) | 96.4 |
| Depression | 93.8 | 96.9 (96.1-97.6) | 95.3 |
| Normal | 88.2 | 99.5 (99.3-99.7) | 93.5 |
| Personality disorder | 98.3 | 94.4 (90.8-97.5) | 96.3 |
| Stress | 96.1 | 97.2 (95.6-98.6) | 96.6 |
| Suicidal ideation | 96.4 | 98.2 (97.5-98.7) | 97.3 |

^a^95% CIs for recall were obtained by bootstrap resampling of the test set with 10,000 replicates.

LLM: large language model; NLP: natural language processing.
